# Inter-observer variability between general pathologists and a specialist in breast pathology in the diagnosis of lobular neoplasia, columnar cell lesions, atypical ductal hyperplasia and ductal carcinoma in situ of the breast

**DOI:** 10.1186/1746-1596-9-121

**Published:** 2014-06-19

**Authors:** Douglas S Gomes, Simone S Porto, Débora Balabram, Helenice Gobbi

**Affiliations:** 1 Breast Pathology Laboratory, School of Medicine, Federal University of Minas Gerais (UFMG), Av. Professor Alfredo Balena, 190, Belo Horizonte, Minas Gerais 30130-100, Brazil

**Keywords:** Breast cancer, Lobular neoplasia, Columnar cell lesions, Atypical ductal hyperplasia, Ductal carcinoma in situ, Inter-observer variability, Agreement

## Abstract

**Background:**

This study aimed to assess inter-observer variability between the original diagnostic reports and later review by a specialist in breast pathology considering lobular neoplasias (LN), columnar cell lesions (CCL), atypical ductal hyperplasia (ADH), and ductal carcinoma in situ (DCIS) of the breast.

**Methods:**

A retrospective, observational, cross-sectional study was conducted. A total of 610 breast specimens that had been formally sent for consultation and/or second opinions to the Breast Pathology Laboratory of Federal University of Minas Gerais were analysed between January 2005 and December 2010. The inter-observer variability between the original report and later review was compared regarding the diagnoses of LN, CCL, ADH, and DCIS. Statistical analyses were conducted using the Kappa index.

**Results:**

Weak correlations were observed for the diagnoses of columnar cell change (CCC; Kappa = 0.38), columnar cell hyperplasia (CCH; Kappa = 0.32), while a moderate agreement (Kappa = 0.47) was observed for the diagnoses of flat epithelial atypia (FEA). Good agreement was observed in the diagnoses of atypical lobular hyperplasia (ALH; Kappa = 0.62) and lobular carcinoma in situ (LCIS; Kappa = 0.66). However, poor agreement was observed for the diagnoses of pleomorphic LCIS (Kappa = 0.22). Moderate agreement was observed for the diagnoses of ADH (Kappa = 0.44), low-grade DCIS (Kappa = 0.47), intermediate-grade DCIS (Kappa = 0.45), and DCIS with microinvasion (Kappa = 0.56). Good agreement was observed between the diagnoses of high-grade DCIS (Kappa = 0.68).

**Conclusions:**

According to our data, the best diagnostic agreements were observed for high-grade DCIS, ALH, and LCIS. CCL without atypia and pleomorphic LCIS had the worst agreement indices.

**Virtual Slides:**

The virtual slide(s) for this article can be found here: http://www.diagnosticpathology.diagnomx.eu/vs/1640072350119725.

## Abstract (pt)

**Abstract:**

**Introdução**

O objetivo do estudo foi avaliar a variabilidade interobservador entre os diagnósticos do laudo original e após revisão por especialista em patologia mamária considerando as neoplasias lobulares (NL), lesões células colunares (LCC), hiperplasia ductal atípica (HAD) e carcinoma ductal in situ (CDIS) em biopsias mamárias.

**Métodos**

Estudo retrospectivo, observacional, do tipo transversal. Um total de 610 casos de espécimes mamários que foram enviados formalmente para consultoria e/ou segunda opinião ao Laboratório de Patologia Mamária da Universidade Federal de Minas Gerais foram analisados entre janeiro de 2005 e dezembro de 2010. A variabilidade interobservador entre o laudo original e o laudo de revisão foi comparada entre os diagnósticos de NL, LCC, HDA e CDIS. A análise estatística foi realizada pelo índice de Kappa.

**Resultados**

Observamos uma concordância fraca para os diagnósticos de alterações de células colunares (ACC; Kappa = 0,38), e hiperplasia de células colunares (HCC; Kappa = 0,32), enquanto uma concordância moderada (Kappa = 0,47) foi observada para o diagnóstico de atipia epitelial plana (AEP). A concordância foi considerada boa para os diagnósticos de hiperplasia lobular atípica (Kappa = 0,62) e carcinoma lobular *in situ* (CLIS; Kappa = 0,66). Entretanto, a concordância foi considerado baixa para o diagnóstico de CLIS pleomórfico (Kappa = 0,22). Concordância moderada foi observada para os diagnósticos de HLA (Kappa = 0,44), CDIS de baixo grau (Kappa = 0,47), CDIS de grau intermediário (Kappa = 0,45) e CDIS microinvasor (Kappa = 0,56). Boa concordância foi observada para o diagnóstico de CDIS de alto grau (Kappa = 0,68).

**Conclusão**

De acordo com nossos dados, as melhores concordâncias diagnósticas foram observadas entre CDIS de alto grau, HLA e CLIS. As LCC sem atipias e o CLIS pleomórfico tiveram os piores índices de concordância.

## Background

Despite advances in the understanding of the molecular biology of breast cancer progression and new molecular markers, the histopathological analysis remains the most widely used diagnostic method of precursor and intraductal proliferative lesions of the breast [1].

Currently, increasing number of breast lesions are discovered during the pre-clinical phase due to the more widespread use of mammography screening and the incorporation of new imaging technologies for the diagnosis of breast cancer. There has also been an increase in the diagnosis of intraductal proliferative and precursor breast lesions, which exhibit uncertain behaviour. These include lobular neoplasia (LN), columnar cell lesions (CCL), atypical ductal hyperplasia (ADH), and ductal carcinoma in situ (DCIS). The differential histologic diagnosis between some of these lesions can be difficult and presents challenges to pathologists; especially those not specialized in breast pathology [2,3].

Reproducibility studies are useful when evaluating the applicability of histological criteria for the classification of breast lesions and when determining the level of agreement amongst pathologists regarding morphological diagnoses. Studies conducted by our group have revealed significant inter-observer variability between the diagnoses made by general pathologists and those made by breast pathology experts in the diagnosis for DCIS and ADH; this discrepancy could have significant therapeutic implications [4,5]. Although there have been various studies on the diagnostic agreement considering DCIS, few studies have analysed the diagnostic agreement considering LN and CCL [6,7].

Our study aimed to assess the frequency of detection rate of precursor lesions and intraductal proliferative lesions, primarily CCL and LN, in breast biopsies sent for consultation as well as the inter-observer variability in the diagnoses made during the original report and a later review by a specialist consultant in breast pathology.

## Methods

A retrospective, observational, cross-sectional study was conducted. Files from the Breast Pathology Laboratory at the School of Medicine of Federal University of Minas Gerais (UFMG), Brazil, were reviewed between January 2005 and December 2010, and 673 cases of breast lesions were identified as having been formally sent for consultation or second opinion. The analysed data were obtained from the original pathologist reports and from the consulting report conducted by a single pathologist (HG) with an expertise on breast pathology. A total of 63 cases were excluded from the analysis; these cases did not have the original reports for comparison or they had insufficient and/or damaged material that prevented the review.

Data were collected through the use of a structured form, and the following items were analysed in both the original report and the review: type of specimen, specialty of the referring physician, and presence of intraductal proliferative lesions (columnar cell lesions [CCL], ADH, and DCIS) and the LN (atypical lobular hyperplasia [ALH], lobular carcinoma in situ [LCIS], and pleomorphic LCIS) associated or not with invasive carcinoma.

The histological classification of LN originally reported by Page *et al*. and adopted by the 2012 World Health Organization (WHO) Classification of Breast Tumours was used [1]. Lobular neoplasia refers to the entire spectrum of atypical epithelial lesions originating in the terminal-duct lobular unit (TDLU) and characterized by a proliferation of generally small, non-cohesive cells. ALH is defined as a filling or expansion of less than 50% of the acini in one or more lobular units by proliferating small, uniform cells. LCIS was defined as a filling and distension of greater than 50% of the acini of a lobular unity and a loss of the residual intracellular lumen [8,9]. The criteria used to diagnose pleomorphic LCIS were those originally described by Eusebi *et al.*, which included the same architectural configuration as LCIS but with increased nuclear pleomorphism, larger nucleoli, with or without comedo necrosis [10].

CCLs are a group of lesions of the terminal ductal-lobular units that are characterized by variably enlarged dilated acini lined by columnar epithelial cells without cytological or architectural atipia [1]. Flat epithelial atypia (FEA), the term adopted by the WHO Classification of Breast Tumours, since 2003, refers to a neoplastic alteration of the TDLUs characterized by replacement of the native epithelial cells by one to several layers of a single epithelial cell type showing low-grade (monomorphic) cytological atypia [1,11]. These lesions differ from those with sufficient architectural and cytological findings for a differential diagnosis of ADH or DCIS. In the present study, we used the diagnostic criteria proposed by Schnitt and Vincent-Salomon, who previously suggested the classification of the FEA group into two groups, CCC and columnar cell hyperplasia (CCH) with atypia according to the number of layers of proliferating epithelial cells (Table 1) [12,13].

**Table 1:** Diagnostic criteria for columnar cell lesions used in the present study

	**Columnar cell change**	**Columnar cell hyperplasia**	**Columnar cell change with atypia**	**Columnar cell hyperplasia with atypia**
			**Flat epithelial atypia**	
**Topography**	Terminal duct-lobular unit with variable dilation	Terminal duct-lobular unit with variable dilation	Terminal duct-lobular unit with variable dilation	Terminal duct-lobular unit with variable dilation
**Architecture**	1 or 2 cell layers	Cell stratification greater than 2 layers, complex cellular configurations are not present	1 or 2 cell layers	Cellular stratification of more than 2 layers, complex cell configurations are not present
**Cytology**	Columnar cells with ovoid to elongated nuclei orientated perpendicular to the basal membrane; nucleolus absent or inconspicuous.	Columnar cells with ovoid to elongated nuclei orientated perpendicular to the basal membrane; “hobnail” cells might appear with absent or inconspicuous nuclei.	Cytological atypia present (usually low-grade); the cells resemble tubular carcinoma. Mitoses are uncommon.	Cytological atypia present (usually low-grade); the cells resemble tubular carcinoma. Mitoses are uncommon.
**Apical decapitation**	Often present, not usually prominent.	Often present, might be exaggerated.	Often present, might be exaggerated.	Often present, might be exaggerated.
**Intraluminal secretions**	Might be present but are not usually prominent.	Might be present and prominent.	Might be present and prominent.	Might be present and prominent.
**Calcifications**	Might be present	Usually present, might be psammomatous.	Usually present, might be psammomatous.	Usually present, might be psammomatous.

Adapted from Schnitt and Vincent-Salomon [12], Fraser *et al*. [13], Tavassoli, & Devilee [11].

ADH was defined as a proliferation of regularly distributed monomorphic cells to form regular, uniform, and circular secondary lumens. These lesions are small, and the cells have 2 partial ducts or “spaces” involved, and are less than 2 mm in size. DCIS is characterised as an epithelial proliferation of atypical cells with 2 complete “spaces” or ducts involved or are more than 2 mm in overall size. The criteria of Elston and Ellis were used to diagnose DCIS with microinvasion [14], which are recognised by predominant DCIS as well as the infiltration of neoplastic cells beyond the basal membrane of the unspecialised or extralobular connective tissue up to 1 mm in size. The histological grades (low, moderate, and high) of DCIS were determined after considering the grade of nuclear atypia as well as the presence and extension of necrosis according to the criteria of Lagios [15]. Currently, the Breast Pathology Laboratory uses the new WHO (2012) Classification for Breast Tumours as a diagnostic reference [1].

To tabulate the data, cases with more than one type of breast lesion were classified according to the lesion with the greatest risk or potential to develop into a carcinoma. For LN, the risk classification was as follows: pleomorphic LCIS > LCIS > ALH; for CCL: CCH atypia > CCC atypia > CCH > CCC; and for DCIS: microinvasive DCIS > high-grade DCIS > intermediate-grade DCIS > low-grade DCIS. Cases of DCIS for which the grade had not been evaluated during the original report or the review were classified as unspecified (US).

The SPSS program (version 17.0; SPSS Inc., Chicago, IL, USA) was used to analyse the inter-observer variability between the original diagnosis and the histopathological review conducted by the consultant pathologist, using the Kappa index. This index was interpreted according to the following values proposed by Landis and Koch [16]: < 0.20 (bad); 0.21–0.40 (poor); 0.41–0.60 (moderate); 0.61–0.80 (good); and 0.81–1.00 (excellent). The significance level *(p)* was defined as 0.05. This study was approved by the Research Ethic’s Committee of the UFMG.

## Results

A total of 610 cases of breast lesions that had been referred for second opinion and satisfied the inclusion criteria were analysed; of these, 56.9% were breast specimens from segmental mastectomies or quadrantectomies, 29.8% from core-biopsies, 5.9% from mastectomies, and 7.4% were other specimen types. The referring practitioner was specialised in breast surgery in 60% of the cases, in oncology in 5.2% of the cases, and in pathology in 4.1% of the cases. Patients’ ages ranged from 13–94 years with a mean age of 53.6 years (±13.3 years).

**Figure 1:**
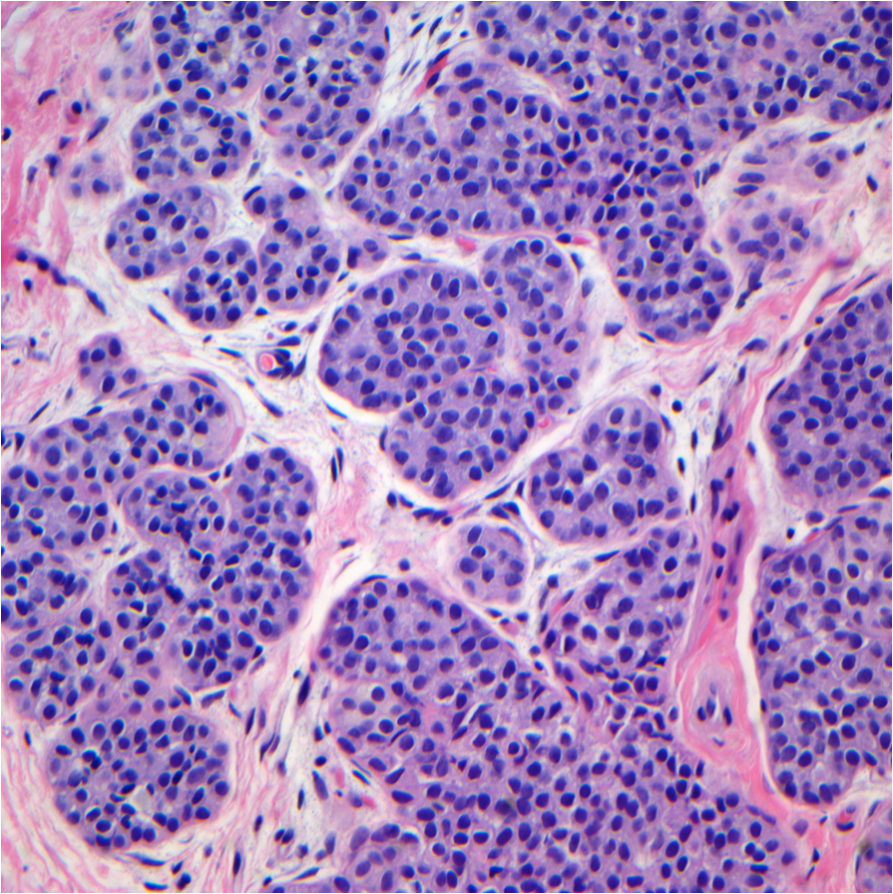
**Lobular neoplasia: this case was originally diagnosed as lobular carcinoma in situ and considered atypical lobular hyperplasia after review.** Less than 50% of lobular units are involved and expanded by uniform cells. (Hematoxylin and eosin; x200).

LN was present in 11.0% (67/610) of the original reports and 11.8% (72/610) of the later reviews. Of 67 cases from the original reports, ALH was present in 25.4%, LCIS in 67.2%, and pleomorphic LCIS in 7.5%. Of the 72 LN cases from the later reviews, ALH was present in 30.6%, LCIS in 63.9%, and pleomorphic LCIS in 5.6% (Table 2; Figure 1). There were good agreements between the original reports and later reviews regarding the diagnoses of ALH (Kappa index = 0.62; [*p* < 0,05]) and LCIS (Kappa index = 0.66; [*p* < 0,05]). However, there was a poor agreement between the diagnoses of pleomorphic LCIS (Kappa index = 0.22; [*p* < 0,05]).

**Table 2:** Diagnostic agreement between the original report and later review of lobular neoplasia

	**LN report review**				
**Original LN report**	**Absent**	**ALH**	**LCIS**	**Pleomorphic LCIS**	**Total**
Absent	521	10	11	1	543
ALH	7	8	2	0	17
LCIS	8	4	31	2	45
Pleomorphic LCIS	2	0	2	1	5
Total	538	22	46	4	610

LN: lobular neoplasia; ALH: atypical lobular hyperplasia; LCIS: lobular carcinoma in situ.

**Figure 2:**
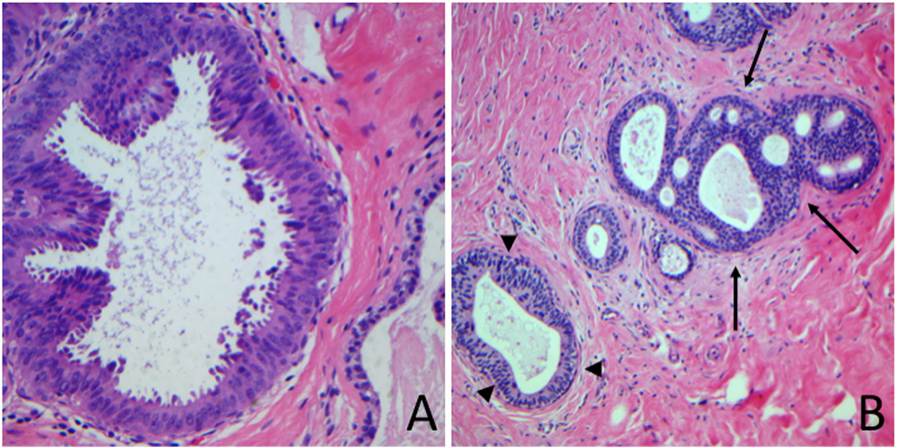
**Diagnostic disagreements between the original diagnosis of flat epithelial atypia and a low-grade ductal carcinoma in situ and the reviewer´s diagnosis. A**: Case originally diagnosed as flat epithelial atypia and considered a columnar cell change without atypia by the reviewer. (Hematoxylin and eosin; x400). **B**: Case diagnosed by the reviewer as atypical ductal hyperplasia (arrows) adjacent to columnar cell change without atypia (arrowheads) and originally considered by the referral pathologist a low-grade ductal carcinoma in situ. (Hematoxylin and eosin; x100).

CCL were present in 14.4% (88/610) of the original reports and 25.1% (153/610) of the reviews. Of 88 cases from the original reports, CCC were present in 64.8%, CCH in 12.5%, CCC with atypia in 8.0%, and CCH with atypia in 14.8% of the cases. Of the 153 cases from the reviews, CCC were present in 74.5%, CCH in 9.8%, CCC with atypia in 6.5%, and CCH with atypia in 9.2% of the cases (Table 3; Figure 2). There were weak diagnostic agreements between the original report and later review for CCC (Kappa index = 0.38; [*p* < 0,05]), CCH (Kappa index = 0.32). The agreement was moderate (Kappa index = 0.47; [*p* < 0,05]) between the diagnoses of FEA (CCC with atypia and CCH with atypia).

**Table 3:** Diagnostic agreement between the original report and later review of columnar cell lesions

	**CCL report review**					
**Original CCL report**	**Absent**	**CCC**	**CCH**	**CCC with atypia**	**CCH with atypia**	**Total**
Absent	437	66	10	2	7	522
CCC	16	36	1	3	1	57
CCH	2	5	3	1	0	11
CCC with atypia	1	3	1	2	0	7
CCH with atypia	1	4	0	2	6	13
Total	457	114	15	10	14	610

CCL: columnar cell lesions; CCH: columnar cell hyperplasia; CCC: columnar cell change.

**Figure 3:**
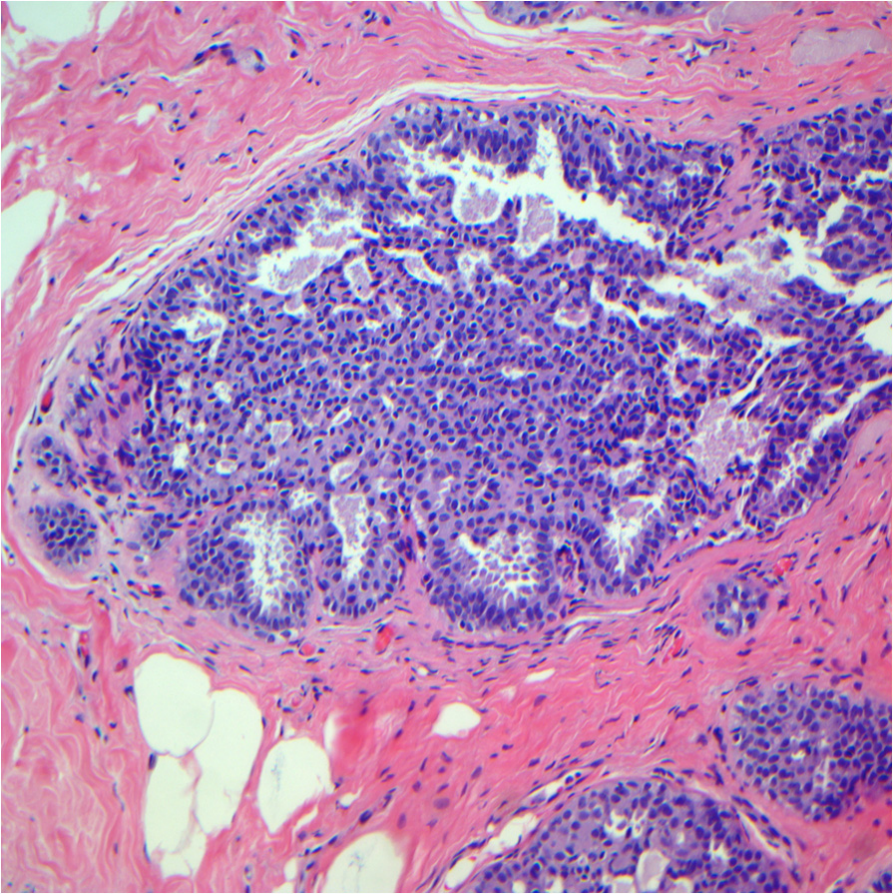
**Case originally diagnosed as atypical ductal hyperplasia and as usual ductal hyperplasia after review.** Note the epithelial cells displaying a haphazard orientation, and the presence of slit-like secondary lumina peripherally located. (Hematoxylin and eosin; x100).

ADH was present in 12.1% (74/610) of the original reports and 8.4% (51/610) of the later reviews (Table 4; Figure 2B and Figure 3). There was a moderate agreement between the original reports and later reviews regarding the diagnosis of ADH (Kappa index = 0.44; [*p* < 0,05]). Of the 74 cases of ADH present in the original reports, in 41.9% (31/74) the reviewer confirmed the diagnosis of ADH. In 58.1% (43/74) cases the ADH was over-diagnosed, these, 58.1% (25/43) the reviewer downgrade diagnosis for usual ductal hyperplasia, in 14.0% (6/43) the diagnosis was increased to DCIS, and 27.8% (12/43) could not evaluate this information only by the reports.

**Table 4:** Diagnostic agreement between the original diagnosis of atypical ductal hyperplasia and the reviewer´s diagnosis

	**Reviewer´s diagnosis of ADH**		
**Original diagnosis of ADH**	**Absent**	**ADH**	**Total**
Absent	516	20	536
ADH	43	31	74
Total	559	51	610

ADH: atypical ductal hyperplasia.

The DCIS frequencies in the original reports and later reviews were 37.7% (230/610) and 39.0% (238/610), respectively. Of 230 DCIS cases from the original reports, low-grade DCIS was present in 25.6%, intermediate-grade DCIS in 23.0%, high-grade DCIS in 39.1%, DCIS-US in 10.9%, and microinvasive DCIS in 1.3%. Of the 238 DCIS cases encountered during later review, low-grade DCIS was present in 26.5%, intermediate-grade DCIS in 20.6%, high-grade DCIS in 44.9%, DCIS-US in 5.9%, and microinvasive DCIS in 2.1% (Table 5). Good diagnostic agreement was observed between the original reports and later reviews for high-grade DCIS (Kappa index = 0.68; [*p* < 0,05]). However, moderate diagnostic agreement was observed for low-grade DCIS (Kappa index = 0.47; [*p* < 0,05]), intermediate-grade DCIS (Kappa index = 0.45; [*p* < 0,05]), and microinvasive DCIS (Kappa index = 0.56; [*p* < 0,05]).

**Table 5:** Diagnostic agreement between the original report and later review of DCIS

	**DCIS report review**						
**Original DCIS report**	**Absent**	**LG DCIS**	**IG DCIS**	**HG DCIS**	**US DCIS**	**MIC DCIS**	**Total**
Absent	333	16	9	19	3	0	380
LG DCIS	16	32	9	0	2	0	59
IG DCIS	8	7	23	12	1	1	53
HG DCIS	6	3	5	72	2	2	90
US DCIS	8	5	3	3	6	0	25
MIC DCIS	0	0	0	1	0	2	3
Total	372	63	49	107	14	5	610

DCIS: ductal carcinoma in situ; LG DCIS: low-grade carcinoma in situ; IG DCIS: intermediate grade carcinoma in situ; HG DCIS: high-grade carcinoma in situ; US DCIS: unspecified carcinoma in situ; MIC DCIS: microinvasive carcinoma in situ.

## Discussion

In this study, we analysed the LN, CCL, ADH and DCIS diagnostic agreements and reproducibility between general pathologists and a specialist pathologist with training and experience in breast pathology in cases received in consultation for a second opinion.

The importance of LN morphological classification is attributed to the Page group and collaborators, who correlated lobular lesion extension with patient evolution. The risks of developing invasive carcinomas were calculated as 4–5% for ALH and 8–11% for LCIS [8,9]. Eusebi *et al*. described the pleomorphic variant of LCIS; this variant features the same architectural arrangement as LCIS but exhibits marked nuclear pleomorphism and abundant cytoplasm, more evident nucleoli, and possible areas of comedo necrosis and microcalcifications either with or without apocrine characteristics [10]. Given the morphological characteristics in association with the fact that the imunohistochemical profile of pleomorphic LCIS is more likely oestrogen receptor-negative and HER-2-positive along with a higher Ki-67 proliferation index that classic LCIS, these lesions have been correlated with a more aggressive biological behavior than that of classic LCIS; however, epidemiological studies to prove this assumption are lacking [1,10]. Recently, molecular analyses of synchronous LCIS and both classic ILC type and the pleomorphic variant of invasive lobular carcinoma demonstrated similarities in the genomic profiles [17]. LCIS is considered both a risk factor and a non-obligate precursor lesion for subsequent invasive carcinomas in either breast, of either ductal or lobular type, but only a minority of women actually develop invasive breast cancer after a long-term follow up [1].

In our study, a good diagnostic agreement was observed between the reports from generalist pathologists and a breast pathology specialist regarding the diagnoses of ALH (Kappa index = 0.62) and LCIS (Kappa index = 0.66). Our data are similar to those of Fitzgibbons, who analysed the responses of 2,952 pathologists to clinical cases from The College of American Pathologists Performance Improvement Program in Surgical Pathology [6]. That study analysed the agreement (%) regarding the diagnosis of ALH; 58% of the pathologists correctly diagnosed ALH, whereas 17% diagnosed LCIS. When LN (ALH and LCIS) cases were assessed together, the agreement rate was 74%. However, there were other conflicting diagnoses for these cases, including ADH (14%), DCIS (1.4%), and usual ductal hyperplasia (10%) [6]. In fact, the differential diagnosis of LN and intraductal proliferative lesions can be difficult, especially when concerning classic LN versus low-grade solid DCIS, and pleomorphic LCIS versus high-grade DCIS [2,18]. Despite the low number of pleomorphic LCIS cases in our study, the inter-observer agreement was poor (Kappa index = 0.22). The immunophenotypic criteria of E-cadherin, β-catenin, and p120-catenin expression in combination with the careful identification of cytological and architectural alterations are useful tools in the morphological classification of these lesions [18,19].

The correct diagnosis of LN cases will affect the treatment options and counseling. Patients with LN are at risk of developing invasive ipsilateral and contralateral breast carcinomas. For this reason, most patients diagnosed with LN are clinically monitored, and tamoxifen might be administered as a prophylactic therapy against the development of invasive carcinomas [20]. In very specific cases in which there are other associated risk factors, a bilateral prophylactic mastectomy might be offered [21]. The management of LN after core-biopsy diagnoses remains controversial. Complete excision of the lesion is recommended in patients who have been diagnosed with various forms of LCIS; however, the current evidence does not support the routine excision of conventional LCIS diagnosed via core-biopsy in cases with a clinical-radiological correlation and in which suspected area on the image has been properly sampled [1,2].

CCL and FEA are a group of breast lesions whose diagnostic criteria were defined only in recent years [12]. With the widespread use of mammography screening, CCLs have often been identified in breast biopsies and are present in as many as half of the biopsies performed for microcalcifications detected via mammography. Recent studies have shown that flat epithelial atypia shares genetic similarities with ADH, low-grade DCIS, and tubular carcinomas, suggesting that these lesions act as precursors of invasive, low-grade carcinomas [22].

Despite advances in genetic studies of these lesions, few studies have assessed the diagnostic reproducibility of CCL amongst pathologists. In our study, we observed weak diagnostic agreements regarding CCC and CCH between generalist pathologists and a breast pathology specialist. When we assessed FEA, the agreement was moderate with better agreement for lesions with more pronounced atypia. Our data differed from those of O’Malley *et al*. [7], who observed excellent agreement (Kappa index = 0.83) regarding the diagnoses of CCL without atypia and FEA. The agreement was better when detecting the absence of FEA (92.8%) than when confirming its presence (90.4%). However, in contrast to our study, the agreement was assessed in selected cases and amongst pathologists experienced in breast pathology using images of the cases with pre-established diagnostic criteria [7]. Haupt *et al*. analysed the diagnostic agreement regarding previously selected CCL cases between residents and fellows both before and after conducting a tutorial on the diagnostic criteria of CCL. Before conducting the tutorial, the diagnostic agreement of FEA was weak (Kappa index = 0.39); after the training, there was a statistically significant increase in the ability to recognise FEA (Kappa index = 0.60) [23]. A similar study was conducted by Tan *et al*., who analysed the diagnostic agreement of CCL amongst pathologists from the same department after a tutorial; the agreement obtained varied from weak to moderate (Kappa index range = 0.44–0.71) between the group of pathologists and the pathologist tutor [24].

The clinical importance of a correct diagnosis of FEA is that these lesions often coexist alongside LN, ADH, low-grade DCIS, and low-grade invasive carcinomas such as tubular carcinomas [22,25]. In a recent meta-analysis, Verschuur-Maes *et al*. analysed 24 studies that reported the presence of carcinoma in situ after diagnosing CCL in needle biopsies. DCIS underestimation rates of 1.5%, 9%, and 20% were observed in cases of CCL without atypia, CCL with atypia, and CCL associated with ADH, respectively [26]. However, given the limitations of the studies and the large variation in the time of follow-up after the initial biopsy, the WHO Classification of Breast Tumours (2012) notes that it remains uncertain whether the indication of surgical excision is necessary after a diagnosis of FEA via core needle biopsy. Radiological and pathological correlations are recommended to determine the subsequent procedure [1]. Moreover, epidemiological studies such as that conducted by Boulos *et al*. have revealed that CCL are associated with only a relative 1.55-fold risk of developing invasive carcinomas in subsequent years; however, the risk associated with these lesions is not entirely independent of the risk associated with other concomitant proliferative lesions [27].

ADH and DCIS comprise 10% and 15–20%, respectively, of all breast lesions detected using mammography screening. The relative risk of developing invasive carcinoma ranges from 4 to 5-fold among patients with a diagnosis of ADH and from 8- to 10-fold among patients with DCIS [1,28]. However, the histopathological diagnoses of these intraductal proliferative lesions may be difficult, and the inter- and intra-observer agreements between the pathologists differ [5,29,30]. This diagnostic inconsistency could be primarily a result of the morphological criteria used to classify and appropriately select the diagnostic fields [30,31]. The correct diagnosis and the differentiation between ADH and DCIS have repercussions for the treatment of these lesions. When diagnosed via core biopsy, ADH lesions must be completely removed to search for DCIS in the excision specimen to avoid a missed detection of an invasive component. In cases in which the diagnosis of ADH was upheld after an extended biopsy, no further treatment is necessary. However, given the greater risk of progressing to an invasive carcinoma, DCIS has been treated by complete excision of the lesion with free margins, with complementary radiotherapy in cases for which breast-conserving surgery has been performed, and the use of tamoxifen as a prophylaxis against local recurrence [32].

In our study, the diagnostic agreements between the original reports and later reviews were moderate for ADH (Kappa index = 0.44), low-grade DCIS (Kappa index = 0.47), intermediate-grade DCIS (Kappa index = 0.45), and microinvasive DCIS (Kappa index = 0.56). Elston *et al*. analysed the level of inter-observer agreement in the diagnoses of ADH and DCIS amongst 23 pathologists who used pre-defined diagnostic criteria. In this study, the Kappa indices were considered poor (0.35) for ADH and good (0.78) for DCIS. However, when DCIS cases were stratified by histological grades, significant variations were observed in the inter-observer diagnoses, as the Kappa indices were 0.51 for low-grade DCIS, 0.19 for intermediate-grade DCIS, and 0.41 for high-grade DCIS [31]. However, in our study, we obtained a better agreement for the diagnosis of high-grade DCIS (Kappa index = 0.68). Similar findings have been described by other authors that conducted studies on the inter-observer variability with regard to the nuclear grade of DCIS. Sneige *et al*. evaluated the inter-observer variability among six pathologists who assessed 125 cases of DCIS and observed a better diagnostic agreement for high-grade DCIS [33,34]. The nuclear grade of DCIS is an important factor when determining the therapeutic approach because high nuclear-grade lesions are associated with a poor prognosis and are often associated with local recurrence and/or progression into invasive lesions, a greater chance of metastasis, and greater required care during local surgical procedures [4,28].

Various strategies have been used in an attempt to improve the diagnoses of ADH and DCIS, including a review of the diagnostic criteria and continuing education programs [33]. Recently, Jain *et al*. revealed that the agreement between nine pathologists regarding the diagnosis of ADH was poor (Kappa index = 0.34) and that an immunohistochemical analysis of cytokeratin (5, 14, 7 and 18) and p63 protein expression significantly improved the level of agreement among pathologists (Kappa index = 0.5) [30]. The influence of automated methods of interpretation [35] and the use of telepathology and virtual slides has been evaluated to improve the accuracy of diagnosis and as a tool for education, quality control, and second opinion in pathology [36,37].

As our study was retrospective, it includes some limitations. Proliferative lesions may or may not be associated with invasive carcinomas, a factor that might cause the generalist pathologist to underreport a case when faced with a more aggressive diagnosis. Although our data were based on pathological reports, upon assessing the aims of this study, we believe that this methodology is similar to that used in clinical practice.

Our study was the first to assess the diagnostic agreement regarding CCL and LN in cases that were sent for consultation according to reports by generalist pathologists. Interestingly, in our series, a total of 60% of cases were sent for consultation by breast surgeons and 5.2% of cases were sent by oncologists. Only 4.1% of cases were sent for consultation by pathologists. We believe that formal requests for second opinions regarding precursor and borderline breast lesions should be encouraged, especially amongst general pathologists, with the aim of reducing errors in diagnosis and thereby assuring appropriate therapeutic conduct and guaranteeing patient safety [4,5].

## Conclusions

Our findings show a low degree of inter-observer diagnostic agreement between generalist pathologists and a specialist in breast pathology with regard to CCL without atypia and pleomorphic LCIS, moderate agreement for FEA, ADH, and low-grade, intermediate, and microinvasive DCIS, and good agreement for high-grade DCIS, ALH, and LCIS. We believe that the use of standardised diagnostic criteria and specific training in breast pathology might improve the reproducibility of these diagnoses, thereby improving the reliability of the pathological reports in the definition of the best therapeutic approach for each patient.

## Abbreviations

ADH: Atypical ductal hyperplasia; ALH: Atypical lobular hyperplasia; CCC: Columnar cell change; CCH: Columnar cell hyperplasia; CCL: Columnar cell lesions; DCIS: Ductal carcinoma in situ; FEA: Flat epithelial atypia; LCIS: Lobular carcinoma in situ; TDLU: Terminal-duct lobular unit LN: lobular neoplasia; UFMG: Federal University of Minas Gerais; WHO: World Health Organization.

## Competing interests

The authors declare that they have no competing interests.

## Authors’ contributions

DSG conceived the study, and drafted the manuscript. SSP participated in the design of the study. DB performed the statistical analysis, and drafted the manuscript. HG participated in design and coordination of the study, participated in the histological review, and drafted and reviewed the manuscript. All authors have read and approved the final manuscript.
